# Inherited Chromosomally Integrated Human Herpesvirus 6 Genomes Are Ancient, Intact, and Potentially Able To Reactivate from Telomeres

**DOI:** 10.1128/JVI.01137-17

**Published:** 2017-10-27

**Authors:** Enjie Zhang, Adam J. Bell, Gavin S. Wilkie, Nicolás M. Suárez, Chiara Batini, Colin D. Veal, Isaac Armendáriz-Castillo, Rita Neumann, Victoria E. Cotton, Yan Huang, David J. Porteous, Ruth F. Jarrett, Andrew J. Davison, Nicola J. Royle

**Affiliations:** a Department of Genetics, University of Leicester, Leicester, United Kingdom; b MRC-University of Glasgow Centre for Virus Research, Glasgow, United Kingdom; c Department of Health Sciences, University of Leicester, Leicester, United Kingdom; d Generation Scotland, Centre for Genomic and Experimental Medicine, Institute of Genetics and Molecular Medicine, University of Edinburgh, Edinburgh, United Kingdom; University of California, Irvine

**Keywords:** human herpesvirus 6, telomere, integration, ciHHV-6, molecular dating, Generation Scotland

## Abstract

The genomes of human herpesvirus 6A (HHV-6A) and HHV-6B have the capacity to integrate into telomeres, the essential capping structures of chromosomes that play roles in cancer and ageing. About 1% of people worldwide are carriers of chromosomally integrated HHV-6 (ciHHV-6), which is inherited as a genetic trait. Understanding the consequences of integration for the evolution of the viral genome, for the telomere, and for the risk of disease associated with carrier status is hampered by a lack of knowledge about ciHHV-6 genomes. Here, we report an analysis of 28 ciHHV-6 genomes and show that they are significantly divergent from the few modern nonintegrated HHV-6 strains for which complete sequences are currently available. In addition, ciHHV-6B genomes in Europeans are more closely related to each other than to ciHHV-6B genomes from China and Pakistan, suggesting regional variation of the trait. Remarkably, at least one group of European ciHHV-6B carriers has inherited the same ciHHV-6B genome, integrated in the same telomere allele, from a common ancestor estimated to have existed 24,500 ± 10,600 years ago. Despite the antiquity of some, and possibly most, germ line HHV-6 integrations, the majority of ciHHV-6B (95%) and ciHHV-6A (72%) genomes contain a full set of intact viral genes and therefore appear to have the capacity for viral gene expression and full reactivation.

**IMPORTANCE** Inheritance of HHV-6A or HHV-6B integrated into a telomere occurs at a low frequency in most populations studied to date, but its characteristics are poorly understood. However, stratification of ciHHV-6 carriers in modern populations due to common ancestry is an important consideration for genome-wide association studies that aim to identify disease risks for these people. Here, we present full sequence analysis of 28 ciHHV-6 genomes and show that ciHHV-6B in many carriers with European ancestry most likely originated from ancient integration events in a small number of ancestors. We propose that ancient ancestral origins for ciHHV-6A and ciHHV-6B are also likely in other populations. Moreover, despite their antiquity, all of the ciHHV-6 genomes appear to retain the capacity to express viral genes, and most are predicted to be capable of full viral reactivation. These discoveries represent potentially important considerations in immunocompromised patients, in particular in organ transplantation and in stem cell therapy.

## INTRODUCTION

Given the complex roles that human telomeres play in cancer initiation and progression and in ageing (1, 2), it is remarkable that the genomes of human herpesviruses 6A (HHV-6A) and HHV-6B (species Human betaherpesvirus 6A and Human betaherpesvirus 6B) can integrate and persist within them (3). Human telomeres comprise double-stranded DNA primarily composed of variable lengths of (TTAGGG)_*n*_ repeats and terminated by a 50- to 300-nucleotide (nt) 3′ single-strand extension of the G-rich strand. Telomeres, bound to a six-protein complex called shelterin, cap the ends of chromosomes and prevent inappropriate double-strand break repair. They also provide a solution to the “end replication problem” via the enzyme telomerase (4–6).

**FIG 1:**
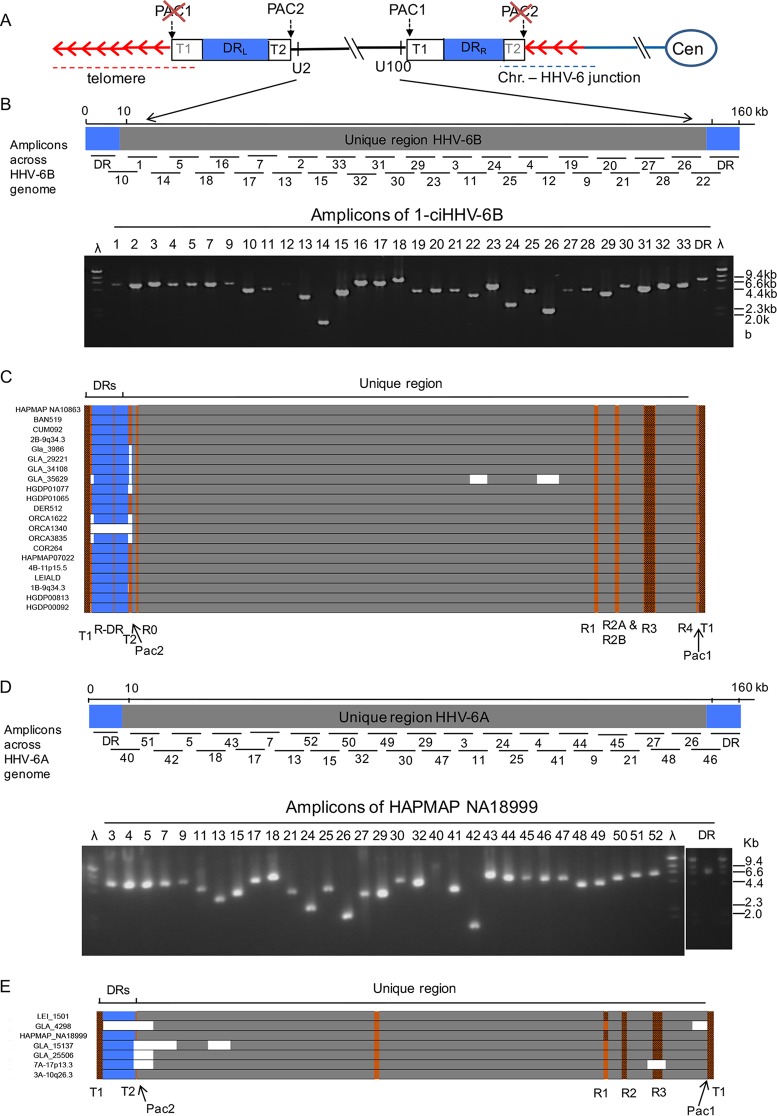
Approach to sequencing ciHHV-6 genomes. (A) Diagram showing the organization of the HHV-6 genome following integration of a single full-length copy into a telomere. The chromosome and centromere (Cen) are represented by blue lines and an oval. The telomere repeats are represented by red arrows. The telomere, encompassing DR_L_-T1, is indicated by the red dashed line. The junction between the chromosome and the HHV-6 genome, encompassing telomere repeats and DR_R_-T2, is indicated by the dashed blue line. DR_L_ and DR_R_ are shown as blue boxes. (B) Distribution of numbered PCR amplicons across the HHV-6B genome and example gel of PCR products generated from 1-ciHHV-6B. (C) Sequence coverage for individual ciHHV-6B genomes. Each ciHHV-6B genome is shown as a single DR (blue box) that was covered by amplicons from DR_L_ and DR_R_ and with U (gray box). Gaps in the coverage caused by loss of individual amplicons at the amplicon-pooling stage are shown in white. Tandem-repeat regions that were fully sequenced by either Illumina NGS or by the Sanger method are shown in orange. Tandem-repeat regions (e.g., T1 and R3 in HHV-6B) that were too long to be sequenced fully are shown as brown boxes. (D) Distribution of numbered PCR amplicons across the HHV-6A genome and example gel of products generated from HAPMAP NA18999. (E) Sequence coverage for ciHHV-6A genomes, using the same color coding as in panel C.

The double-stranded DNA genomes of HHV-6A and HHV-6B consist of a long unique (U) region (143 to 145 kb) including many functional open reading frames (ORFs) (U2 to U100), flanked by identical left and right direct repeats (DR_L_ and DR_R_; 8 to 10 kb) including two ORFs (DR1 and DR6). Each DR also contains near its ends two variable regions of telomere-like repeat arrays (T1 and T2) (7, 8), terminated by the viral-genome-packaging sequences (PAC1 and PAC2, respectively) (9, 10). Telomeric integration by HHV-6A or HHV-6B (yielding chromosomally integrated HHV-6 [ciHHV-6]) results in loss of the terminal PAC2 sequence at the fusion point between the telomere and DR_R_-T2 (11) and loss of the DR_L_-PAC1 sequence at the other end of the integrated viral genome when the DR_L_-T1 degenerate telomere-like repeat region becomes part of a newly formed telomere (Fig. 1A) (12).

Once the HHV-6 genome has integrated in the germ line, it can be passed from parent to child, behaving essentially as a Mendelian trait (inherited ciHHV-6) (13–16). The telomere carrying the ciHHV-6 genome shows instability in somatic cells, which can result in the partial or complete release of the viral genome as circular DNA (12, 17, 18). This could represent the first step toward viral reactivation, and in this respect, telomeric integration may be a form of HHV-6 latency. To date, reactivation of ciHHV-6 has been demonstrated *in vivo* in two settings: first, in a child with X-linked severe combined immunodeficiency who was also a carrier of inherited ciHHV-6A (19), and second, upon transplacental transmission from two ciHHV-6 carrier mothers to their noncarrier babies (20). Recently, it has been shown that ciHHV-6 carriers bear an increased risk of angina pectoris (21), although it is not known whether this arises from viral reactivation, a deleterious effect on the telomere carrying the viral genome, or some other mechanism.

A small proportion of people worldwide are carriers of inherited ciHHV-6A or -6B, but very little is known about the HHV-6 genomes that they harbor, although they may influence any associated disease risk. To investigate ciHHV-6 genomic diversity and evolution, the frequency of independent germ line integrations, and the potential functionality of the integrated viral genomes, we analyzed 28 ciHHV-6 genomes. We discovered that ciHHV-6 genomes are more similar to one another than to the few sequenced reference HHV-6 genomes from nonintegrated viruses. This is particularly marked among the ciHHV-6B genomes from Europeans. We also found that a subset of ciHHV-6B carriers from England, Orkney, and Sardinia are most likely descendants of a single ancient ancestor. Despite the apparent antiquity of some, possibly most, ciHHV-6 genomes, we concluded that the majority contain a full set of intact HHV-6 genes and therefore in principle retain the capacity to generate viable viruses.

## RESULTS

### Selection of ciHHV-6 carriers and sequence analysis of viral genomes.

To investigate sequence variation among ciHHV-6 genomes, 28 samples were selected for analysis: 7 with ciHHV-6A (including LEI-1501) (18) and 21 with ciHHV-6B (Table 1). The selected samples were identified in the various populations screened (Table 2) and included additional individuals from the London area (16), Scotland and the north of England (22), the Leicester area of England (18), and the Generation Scotland: Scottish Family Health Study (GS:SFHS) (R. F. Jarrett, unpublished data). The chromosomal locations of ciHHV-6 genomes, determined by fluorescent *in situ* hybridization (FISH), were available for some samples (16, 18). For other samples, the junction between the viral DR8 sequence (a noncoding region near one end of the DR) and the chromosome subtelomeric region was isolated by PCR and sequenced (discussed below). Integration of each ciHHV-6 genome was confirmed by detection of a telomere at DR_L_-T1 using single-telomere length analysis (STELA) (12) or by detection of at least one copy per cell using droplet digital PCR (22, 23).

**TABLE 1:** Samples from individuals with ciHHV-6 selected for viral genome sequencing

Sample	Accession no.	ciHHV-6	Integration site^*a*^	Population or country
LEI-1501^*b*^	KT355575	A	19q	Leicester area (England)
HAPMAP NA18999	KY316047	A		Japan
3A-10q26.3^*c*^	KY316049	A	10q26.3 and junction isolated by PCR	Southeast England
GLA_4298^*d*^	KY316056	A		Newcastle (England)
GLA_15137^*e*^	KY316055	A		Scotland
GLA_ 25506^*e*^	KY316054	A		Scotland
7A-17p13.3^*c*^	KY316048	A	17p13.3	Southeast England
HGDP00092	KY316037	B		Balochi (Pakistan)
HGDP00813	KY316036	B		Han (China)
HGDP01065	KY316035	B	Junction isolated by PCR	Sardinia (Italy)
HGDP01077	KY316034	B	Junction isolated by PCR	Sardinia (Italy)
HAPMAP NA07022 (CEPH 1340.11)	KY316039	B		Utah Mormon (north European)
HAPMAP NA10863 (CEPH-1375.02)	KY316038	B		Utah Mormon (north European)
4B-11p15.5^*c*^	KY316044	B	11p15.5	Southeast England
BAN519^*f*^	KY316043	B		Banff (Scotland)
COR264^*f*^	KY316042	B		Cornwall (England)
CUM082^*f*^	KY316041	B		Cumbria (England)
DER512^*f*^	KY316040	B	Junction isolated by PCR	Derbyshire (England)
2B-9q34.3^*c*^	KY316045	B	9q34.3	Southeast England
1-ciHHV-6B^*c*^	KY316046	B	Junction isolated by PCR	Southeast England
LEI-ALD	KY316033	B		Leicester area (England)
ORCA1622	KY316031	B		Orkney
ORCA1340	KY316032	B		Orkney
ORCA3835	KY316030	B		Orkney
GLA_3986^*d*^	KY316053	B		Newcastle (England)
GLA_29221^*e*^	KY316052	B		Scotland
GLA_34108^*e*^	KY316051	B		Scotland
GLA_35629^*e*^	KY316050	B		Scotland

a Determined by FISH or amplification of chromosme–ciHHV-6 junctions by PCR.

b LEI-1501 (18).

c ciHHV-6 carriers (16).

d ciHHV-6 carriers (22).

e ciHHV-6 carriers identified in the GS:SFHS.

f Samples from the Population of British Isles study (36).

**TABLE 2:** Summary of populations screened for ciHHV-6^*a*^

Continent	Region or population	No. of samples			
		Total	Total ciHHV-6	ciHHV-6A	ciHHV-6B
Africa	Sub-Saharan Africa	105	0	0	0
	North Africa	29	0	0	0
Europe	North European extraction (CEPH)	136	2	0	2
	British	518	7	1	6
	Orkney	2,194	42^*b*^	0	42
	Italy (including Sardinia)	49	2	0	2
	France	52	0	0	0
	Russia	42	0	0	0
Middle East	Israel	134	2	2	0
South/Central Asia	Pakistan	192	1	0	1
	Uygur (China)	10	0	0	0
East Asia	China	213	1	0	1
	Japan	74	1	1	0
	Others (Siberia and Cambodia)	35	0	0	0
Oceania	Bougainville	17	0	0	0
	New Guinea	11	0	0	0
America	South America	29	0	0	0
	Mexico	35	0	0	0
Total		3,875	58	4	54

a In addition, A. J. Bell, R. F. Jarrett, and colleagues screened the Generation Scotland: Scottish Family Health Study cohort for ciHHV-6 (R. F. Jarrett, unpublished data).

b Frequency, 1.9%.

Each viral genome from ciHHV-6 carriers was sequenced from pooled PCR amplicons (12, 18). Full sets of HHV-6 amplicons were readily generated (Fig. 1; see Table S1 in the supplemental material), demonstrating the robustness of this approach for enriching HHV-6 sequences from ciHHV-6 carriers. The HHV-6 amplicons generated from each carrier had the expected sizes, with variation only in amplicons encompassing repetitive regions (e.g., the DR_R_-T1 region of degenerate telomere-like repeats). This observation indicated that all of the ciHHV-6 genomes are essentially intact, with the exception of the terminal DR_R_-PAC2 and DR_L_-PAC1 sequences lost during integration (Fig. 1A) (11, 12).

The ciHHV-6 genome sequences were determined by short-read next-generation sequencing (NGS), with some verification by the Sanger method. *De novo* assemblies of each genome were generated with few gaps (Fig. 1). The ciHHV-6A genome reported previously by us was included in these analyses (LEI-1501; GenBank accession no. KT355575) (18).

### Sequence similarity is greater among ciHHV-6 genomes than to nonintegrated HHV-6 genomes.

**FIG 2:**
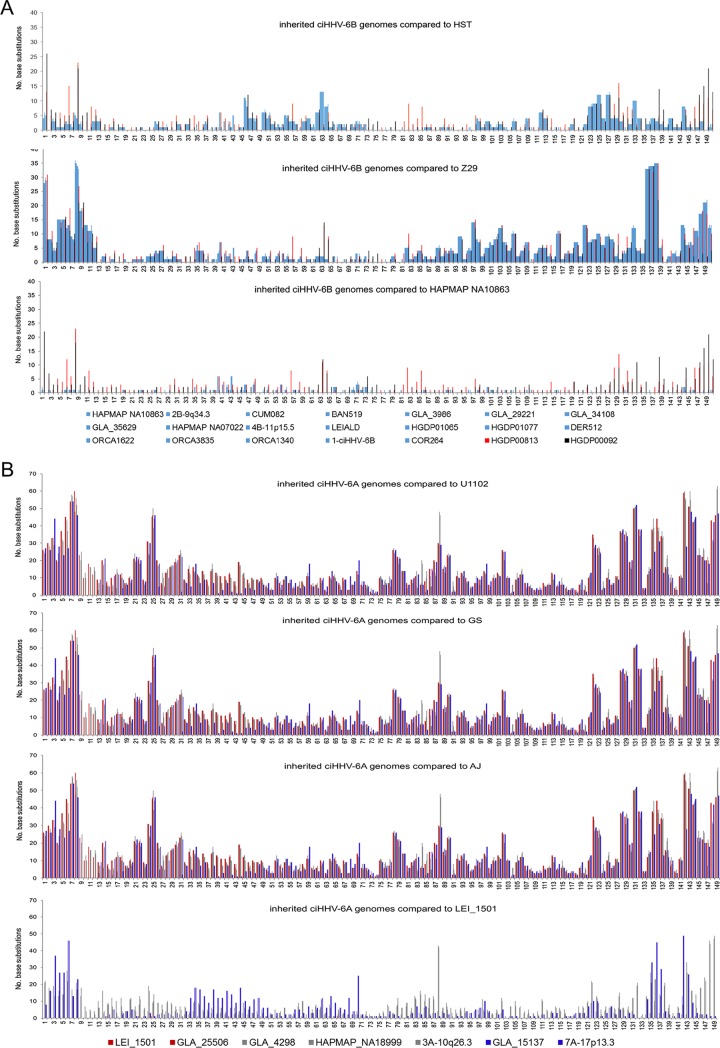
Frequencies of nucleotide substitutions in ciHHV-6 genomes compared to reference viral genomes. (A) Number of substitutions in 1-kb windows for each of the 21 ciHHV-6B genomes in comparison with the HHV-6B strain HST (Japan) and Z29 (Democratic Republic of the Congo) genomes (top and middle, respectively) and the ciHHV-6B genome from HAPMAP NA10863 (bottom). ciHHV-6B genomes from individuals with European ancestry are represented as light-blue lines; ciHHV-6B in HGDP00813 (China), red lines; ciHHV-6B in HGDP00092 (Pakistan), black lines. (B) Number of substitutions in 1-kb windows for each of the 7 ciHHV-6A genomes in comparison with the HHV-6A strain U1102 (Uganda), GS (United States), and AJ (Gambia) genomes (top and middle [two]) and the ciHHV-6A genome in LEI-1501 (bottom). The color key distinguishes the ciHHV-6A genomes. The *x* axes in all the graphs show the HHV-6B and -6A genomes with a single DR (kb 0 to 8) followed by U (kb 9 to 150), as shown in Fig. 1C and E. Variation within the tandem-repeat regions is not shown in the graphs.

Nucleotide substitution frequencies were analyzed across the DR and U regions of the HHV-6B genome (excluding the tandem-repeat regions R-DR, R0, R1, R2, R3, and R4, [Fig. 1]) (9, 24) for each sequenced ciHHV-6B genome in comparison with the two available HHV-6B reference genomes from nonintegrated strains (HST from Japan, GenBank accession no. AB021506 [24], and Z29 from the Democratic Republic of the Congo, GenBank accession no. AF157706 [9]). The ciHHV-6B genomes show different patterns of variation from the reference genomes, with greater divergence from strain Z29 in the distal portion of the U region (kb 120 to 150) and across the DR (kb 1 to 8), reaching a maximum of 35 substitutions per kilobase in these regions (Fig. 2A). Overall, there is less divergence from strain HST, although the frequency of substitutions is higher in part of the U region (kb 45 to 64) than in strain Z29. To assess sequence variation among the ciHHV-6B genomes, comparisons were made using the genome in the European individual HAPMAP NA10863 (CEPH1375.02) as a reference. The substitution frequency is considerably less across the viral genomes for 18/20 ciHHV-6B genomes from individuals with European ancestry, indicating greater similarity among them. Notably, the other two ciHHV-6B genomes, which showed higher substitution frequencies in this comparison, were in individuals from Pakistan and China, HGDP00092 and HGDP00813, respectively (Fig. 2A).

Nucleotide substitution frequencies were also analyzed across each of the seven ciHHV-6A genomes in comparison with three nonintegrated HHV-6A reference genomes (strain U1102 from Uganda [25, 26] [accession no. X83413]; strain GS from the United States (accession no. KC465951.1 [GS1] and KJ123690.1 [GS2] [27, 28], and strain AJ from the Gambia [accession no. KP257584.1] [29]). This analysis showed that the ciHHV-6A genomes have similar levels of divergence from each reference genome from nonintegrated HHV-6A and that divergence is highest across the DR and the distal part of the U region (kb 120 to 149) (Fig. 2B). Comparisons with the ciHHV-6A LEI-1501 genome (18) as a reference also showed greater similarity among the ciHHV-6A genomes, although the substitution frequencies are higher than among European ciHHV-6B genomes, indicating greater diversity among the ciHHV-6A genomes sequenced here (30). Notably, ciHHV-6A in the Japanese individual (HAPMAP NA18999) showed greater divergence from the other ciHHV-6A samples of European origin.

In summary, comparisons of nucleotide substitution frequencies showed that the viral genomes in ciHHV-6B carriers are more similar to each other than they are to reference genomes derived from clinical isolates of nonintegrated HHV-6B from Japan (HST) and the Democratic Republic of the Congo (Z29). The ciHHV-6A genomes are also more similar to each other than they are to the three HHV-6A reference genomes, although this is less pronounced than among the ciHHV-6B genomes.

### Phylogenetic analysis of ciHHV-6 and nonintegrated HHV-6 genomes.

**FIG 3:**
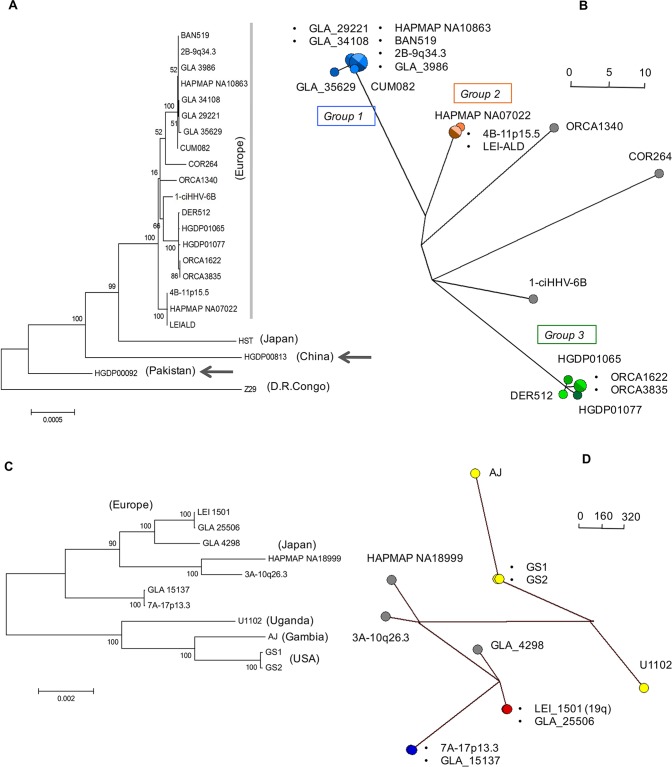
Phylogenetic analysis of ciHHV-6 and reference nonintegrated HHV-6 genomes. (A) Maximum-likelihood phylogenetic tree of 21 ciHHV-6B genomes and two HHV-6B reference genomes (strains HST [Japan] and Z29 [Democratic Republic of the Congo]). A total of 130,412 nucleotides were analyzed, excluding repeat regions and missing amplicons. The scale bar represents 0.0005 substitutions per site. (B) Phylogenetic network generated from the data set used in panel A, but without the HST and Z29 genomes and the ciHHV-6B genomes from HGDP00813 (China) and HGDP00092 (Pakistan). The ciHHV-6B genomes from Europeans in groups 1, 2, and 3 are shown as blue, orange, and green dots, respectively, and the singletons are shown as gray dots. (C) Maximum-likelihood phylogenetic tree of seven ciHHV-6A genomes and four HHV-6A reference genomes (strains U1102 [Uganda], AJ [Gambia], and GS1 and GS2 [United States]; GS1 and GS2 are two versions of strain GS). A total of 117,900 nucleotides were analyzed, excluding repeat regions and missing amplicons. The scale bar represents 0.002 substitutions per site. (D) Phylogenetic network generated from the data set used in panel C. The nonintegrated HHV-6A reference genomes are shown as yellow dots. The closely related ciHHV-6A genomes are shown as pairs of red or blue dots and singletons as gray dots (including one from Japan). The scale bars in the networks (C and D) show the number of base substitutions for a given line length. The dots are scaled, with the smallest dot representing a single individual.

Consistent with the results shown in Fig. 2, phylogenetic analysis of the U regions from 21 ciHHV-6B genomes and the HST and Z29 reference genomes (excluding the DR, the large repeat regions, and missing data shown in Fig. 1) showed that the ciHHV-6B genomes in HGDP00813 from China and HGDP00092 from Pakistan are outliers to the 19 ciHHV-6B genomes from individuals of European descent (Fig. 3A). A phylogenetic network of the ciHHV-6B genomes from individuals with European ancestry showed three clusters of 8, 3, and 5 closely related ciHHV-6B genomes (groups 1, 2, and 3, respectively) (Fig. 3B) and three singletons (ORCA1340, COR264, and 1-ciHHV-6B). Phylogenetic analysis of the DR alone showed that, with the exception of COR264, the European ciHHV-6B samples showed greater similarity to the HST (Japan) reference genome than to the Z29 (Democratic Republic of the Congo) reference genome. However, the DRs in the two non-European ciHHV-6B samples HGDP000813 (China) and HGDP00092 (Pakistan) did not cluster closely with those in the European ciHHV-6B samples, again indicating these ciHHV-6B strains are distinct (Fig. 3A; see Fig. S1 in the supplemental material).

**FIG 4:**
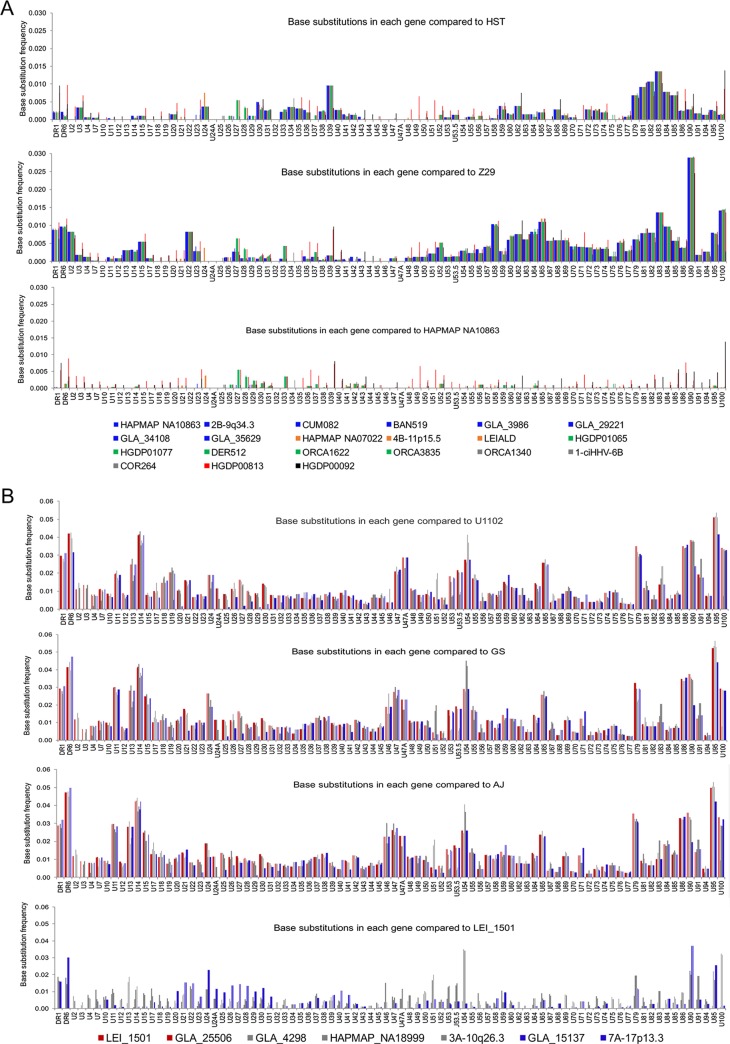
Frequency of nucleotide substitutions in ciHHV-6 genes compared to those in reference viral genomes. (A) Substitution frequencies in each gene are shown for the 21 ciHHV-6B genomes in comparison with HHV-6B strain HST (Japan) and Z29 (Democratic Republic of the Congo) genomes (top and middle, respectively) and the ciHHV-6B genome in European HAPMAP NA10863 (bottom). The color coding shown in the key matches that of the network in Fig. 3B as follows: European group 1, pale-blue lines; European group 2, orange lines; European group 3, green lines; European singletons, gray lines; ciHHV-6B in HGDP00813 from China, red lines; and ciHHV-6B in HGDP00092 from Pakistan, black lines. (B) Substitution frequency in each gene for each of the 7 ciHHV-6A genomes in comparison with the HHV-6A strain U1102 (Uganda), GS (United States), and AJ (Gambia) genomes (top and middle [two]) and the ciHHV-6A genome in European LEI-1501 (bottom). The color key matches that of the network in Fig. 3D. The *x* axes of all the graphs show a single copy of DR1 and DR6, followed by genes found in the U region.

To explore variation only within HHV-6B genes, the frequencies of substitutions in ORFs of each of the 21 ciHHV-6B genomes were compared with those in the HST and Z29 reference genomes and the ciHHV-6B genome in HAPMAP NA10863 (Fig. 4A). The patterns of variation were similar to those observed across the whole genome (Fig. 2A) and consistent with the phylogenetic analysis, showing greater similarity among ciHHV-6B in Europeans and with the subgroups. Phylogenetic analysis of specific genes, which were selected because they show greater sequence variation from the reference genomes or among the ciHHV-6B genomes, generated a variety of trees that were generally consistent with the phylogenetic analysis based on the U region but exhibited less discrimination between samples or groups (Fig. 4; see Fig. S2 in the supplemental material). For example, the phylogenetic tree based on U90 separates the European ciHHV-6B samples from the ciHHV-6B samples from China and Pakistan and from the HST and Z29 reference genomes but does not subdivide the European ciHHV-6B samples.

Phylogenetic analysis of the seven ciHHV-6A genomes and four reference genomes (U1102 [Uganda], AJ [Gambia], and two sequences from GS [United States]) showed a clear separation between the integrated and nonintegrated genomes (Fig. 3C and D), with two pairs of closely related ciHHV-6A genomes (LEI-1501 and GLA_25506; 7A-17p13.3 and GLA_15137). A similar separation of the integrated versus nonintegrated genomes is also evident in the phylogenetic analysis of the DR alone, irrespective of the geographic origin of the individual ciHHV-6A carrier (see Fig. S1 in the supplemental material).

Variation within HHV-6A genes was also explored by plotting the base substitution frequency per ORF for each of the seven ciHHV-6A samples in comparison to the three reference genomes and the ciHHV-6A genome in LEI_1501 (Fig. 4B). The patterns of variation are similar to those observed across the whole genome (Fig. 2B). Phylogenetic analyses of U83, U90, and DR6, selected because they show greater sequence variation, generally support the phylogenetic trees and networks generated from analysis of the U and DR regions (see Fig. S3 in the supplemental material).

Overall, the sequence variation and phylogenetic analyses indicate a divergence between the integrated and nonintegrated HHV-6 genomes but with some differences between HHV-6A and HHV-6B. The ciHHV-6B samples from individuals with European ancestry showed divergence from both the HST (Japan) and Z29 (Democratic Republic of the Congo) reference genomes, although the pattern of divergence varies across the genome. The 21 ciHHV-6B genomes from individuals with European ancestry are more similar to one another than to the ciHHV-6B genomes from China and Pakistan and can be subdivided into distinct groups. There is greater divergence among the seven ciHHV-6A genomes than among the ciHHV-6B genomes, but despite this, two pairs of closely related ciHHV-6A genomes were identified.

From these analyses, we concluded that the three groups of closely related ciHHV-6B genomes and the pairs of ciHHV-6A genomes identified in the phylogenetic networks (Fig. 3B and D, respectively) could represent independent integrations by closely related strains of HHV-6B or HHV-6A. Alternatively, each group might have arisen from a single integration event, with members sharing a common ancestor. Further analyses were undertaken to explore these possibilities.

### Comparison of tandem-repeat regions in ciHHV-6 genomes.

**FIG 5:**
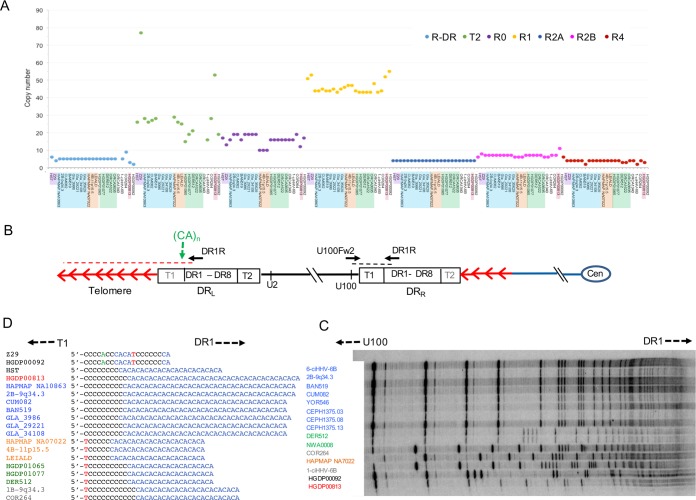
Copy number variation in tandem-repeat loci across the HHV-6B genome. (A) Number of repeat units at loci within the DR (R-DR and DR_L_-T2) and U (R0, R1, R2A, R2B, and R4) regions. Comparisons can be made among the reference nonintegrated HHV-6B strain HST (Japan) and Z29 (Democratic Republic of the Congo) and ciHHV-6B genomes. The sample order along the *x* axis is as follows: HST, Z29 (mauve); European group 1 ciHHV-6B genomes (blue); European group 2 ciHHV-6B genomes (orange); European group 3 ciHHV-6B genomes (green); European singleton ciHHV-6B genomes (no highlight); ciHHV-6B in HGDP00813 from China (red); and ciHHV-6B in HGDP00092 from Pakistan (no highlight). (B) Locations of the PCR amplicons used to analyze the repeat sequences shown in panels C and D. The black dashed line indicates the amplicon generated by the U100Fw2 and DR1R primers that were used for TVR-PCR shown in panel C. The red dashed line indicates STELA products, generated from DR1R, that were used to analyze the (CA)_*n*_ repeat shown in panel D. (C) Distribution of TTAGGG repeats at the distal end of DR_R_-T1 (near U100) in ciHHV-6B genomes. If the repeat array comprises consecutive TTAGGG repeats, a ladder of bands with 6-base periodicity should be present, and the migration distance between the rungs on the ladder should steadily decrease as the separation between the bands is reduced (near the top of the gel, toward DR1). The observed distance between the bands in each track varies between the samples. This shows that the repeat array is not pure (TTAGGG)_*n*_ but includes intervening sequence, most likely degenerate telomere-like repeats. The patterns of repeats can be compared between the tracks to identify samples that share the same repeat distribution at that end of DR_R_-T1. The ciHHV-6B samples are color coded in accordance with groupings identified in Fig. 3: European group 1, blue; group 2, orange; group 3, green; European singletons, gray; ciHHV-6B in HGDP00813 from China, red; ciHHV-6B in HGDP00092 from Pakistan, black. (D) Variation in copy numbers of CA repeats and adjacent 5′ sequence near the start of the ciHHV-6B DR_L_-T1 region. The samples are color coded as in panel C.

Tandem-repeat arrays within the human genome often show length variation as a consequence of changes to the number of repeat units present (copy number variation). The greater allelic diversity in these regions reflects the underlying replication-dependent mutation processes in tandem-repeat arrays, which occur at a higher rate than base substitutions (31). To explore diversity among the ciHHV-6B genomes further, tandem-repeat regions distributed across the viral genome were investigated. The R-DR, R2A, R2B, and R4 repeat regions analyzed (locations are shown in Fig. 1C) showed little or no copy number variation among the ciHHV-6B and nonintegrated reference genomes (Fig. 5A and Table 3). Copy number variation at R1 (location shown in Fig. 1C) was greater but did not show a clear relationship with strains of ciHHV-6B or nonintegrated HHV-6B. Greater copy number variation was detected at the pure array of TTAGGG repeats at DR_L_-T2 (location shown in Fig. 5B), with the largest number of repeats in the HHV-6B Z29 reference genome and ciHHV-6B in HGDP00813 from China (Fig. 5A and Table 3). Notably, the copy number variation observed at R0 (location shown in Fig. 1C) correlates reasonably well with the groups of ciHHV-6B genomes identified in the phylogenetic network (Fig. 3 and 5A and Table 3).

**TABLE 3:** Variation in tandem-repeat regions among ciHHV-6

Parameter	Value^*f*^									
Tandem repeat regions in HHV-6B										
Name	T1	STR (CA)*_n_*	R-DR^*a*^	T2	R0^*a*^	R1	R2A^*a*^	R2B	R3	R4^*a*^
Location		Adjacent to DR-T1	DR	DR	U1	U86	U86–U89	U86–U89	U91–U94	After U100
Length (bp)	6	2	15	6	∼15	12	79	12–15	∼104	64
Unit		CA	NI^*b*^	TTAGGG	NI	NI	NI	NI	NI	NI
**HST**^*c*^	−^*e*^	12	6	26	17	51	4	6	−	6
**Z29**^*c*^	−	1	4	77	13	53	4	8	−	4
HAPMAP NA10863	−	20	5	28	16	44	4	7	−	4
2B-9q34.3	−	20	5	26	19	44	4	7	−	4
CUM082	−	19	5	27	19	45	4	7	−	4
BAN519	−	19	5	28	16	44	4	7	−	4
GLA_3986	−	20	5	−	19	44	4	7	−	2
GLA_29221	−	19	5	−	19	45	4	7	−	4
GLA_34108	−	19	5	−	19	43	4	7	−	4
GLA_35629	−	−	5	−	19	45	4	7	−	4
HAPMAP NA07022	−	11	5	29	10	46	4	6	−	4
4B-11p15.5	−	11	5	26	10	47	4	6	−	4
LEI-ALD	−	11	5	25	10	47	4	6	−	4
HGDP01065	−	10	5	15	16	44	4	7	−	4
HGDP01077	−	10	5	19	16	43	4	7	−	4
DER512	−	10	5	21	16	43	4	7	−	4
ORCA1622	−	−	5	−	16	43	4	7	−	3
ORCA3835	−	−	5	−	16	43	4	7	−	3
ORCA1340	−	−	−	−	16	48	4	6	−	4
1-ciHHV-6B	−	12	5	16	16	43	4	6	−	4
COR264	−	12	9	28	19	44	4	7	−	2
HGDP00813	−	20	3	53	12	52	4	7	−	4
HGDP00092	−	1	2	19	17	55	4	11	−	3
Tandem-repeat regions in HHV-6A										
Name	T1	T2	R5^*d*^	R1	R2	R3				
Location		DR	U41–U42	U86	U86–U89	U91–U94				
Length (bp)	6	6	∼191	∼12	12–18	104–105				
		TTAGGG	NI	NI	NI	NI				
**AJ**^*c*^	−	51	1.7	52	43	8				
**U1102**^*c*^	−	59	1.7	52	102	29				
**GS1/2**^*c*^	−	51	1.7	52	78	8				
LEI-1501	−	14	2.7	−	−	−				
GLA_25506	−	−	2.7	32	−	−				
GLA_4298	−	−	3.7	53	−	−				
HAPMAP NA18999	−	13	1.7	−	−	−				
3A-10q26.3	−	9	1.7	58	−	−				
GLA_15137	−	−	1.7	55	−	−				
7A-17p13.3	−	−	1.7	55	−	−				

a Repeats specific to HHV-6B. The coordinates of R-DR and R4 in HHV-6B strain HST are 5400 to 5489 and 152603 to 152986, respectively.

b NI, repeats are not identical.

c Reference genomes are in boldface.

d Repeat specific to HHV-6A; the coordinates of R5 in HHV-6A strain U1102 are 68124 to 68450; the other repeats are described in references 9 and 24.

e −, analysis was not completed.

f Samples that showed close phylogenetic relationship in Fig. 3 are grouped in the table, with a space between each group.

Similar analysis of repeat regions in the ciHHV-6A genomes was conducted (Table 3). The data suggest that ciHHV-6A genomes have fewer TTAGGG repeats at DR_L_-T2 than the HHV-6A reference genomes. This variation could have been present in HHV-6A strains prior to integration, or deletion mutations that reduced the length of the DR_L_-T2 array may have been favored after integration (12).

To explore variation within the T1 array of degenerate telomere-like repeats in ciHHV-6B genomes, we amplified the DR_R_-T1 region using the U100Fw2 and DR1R primers and investigated the interspersion patterns of TTAGGG and degenerate repeats at the distal end of DR_R_-T1 (near U100) (Fig. 5B) by using modified telomere variant repeat mapping by PCR (TVR-PCR) (32–34). Comparison of the TTAGGG interspersion patterns between the samples showed that the ciHHV-6B genomes clustered into groups that share similar TVR maps in DR_R_-T1 (Fig. 5C). Furthermore, these interspersion patterns differed between the groups and the singleton ciHHV-6B genomes identified in the phylogenetic analyses. Variation around the (CA)_*n*_ simple tandem repeat, located immediately adjacent to DR_L_-T1 (location shown in Fig. 5B), also showed clustering into groups that correlate with the ciHHV-6B phylogenetic analyses (Fig. 3 and 5D and Table 3). Overall, the analyses of tandem-repeat regions in the ciHHV-6B genomes are consistent with the phylogenetic analyses.

### Ancestry of ciHHV-6B carriers in group 3.

**FIG 6:**
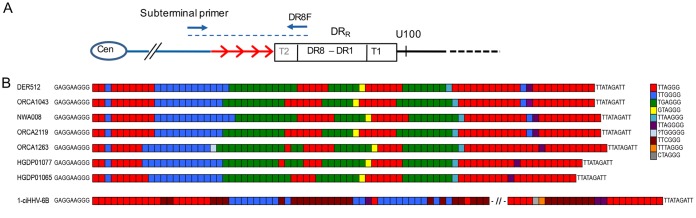
Characterization of ciHHV-6B integration sites. (A) Diagram showing the locations of PCR amplicons used to characterize the chromosome–ciHHV-6B junctions. The red arrows represent TTAGGG and degenerate repeats; blue arrows, primers used to amplify the chromosome–HHV-6 junction; blue dashed line, chromosome junction amplicon used for sequence analysis. (B) Diagram showing the similarity of the TTAGGG and degenerate-repeat (see color key on the right) interspersion patterns in the chromosome–HHV-6 junctions from individuals with group 3 ciHHV-6B genomes (DER512 to HGDP01065 [Fig. 3B]). These interspersion patterns are distinct from that of the chromosome junction fragment isolate from 1-ciHHV-6B (singleton in Fig. 3B). The sequence on the left of the repeats is from the chromosome subtelomeric region, and the sequence on the right is from the ciHHV-6B genome.

The repeat copy number variation observed within and among groups may have arisen before or after telomeric integration of the viral genome. To investigate further how many different integration events may have occurred among the ciHHV-6B carriers, we isolated and sequenced fragments containing the junction between the human chromosome and the ciHHV-6B genome, in addition to using the cytogenetic locations published previously for some samples (Table 1) (16). The junction fragments were isolated by a trial-and-error approach, using PCR between a primer mapping in DR8 in DR_R_ and a variety of primers known to anneal to different subtelomeric sequences (Fig. 6A), including primers that anneal to the subterminal regions of some, but not all, copies of chromosome 17p (17p311 [35] and sub-T17-539 [12]). There was insufficient DNA for analysis from the sequenced ORCA1340 (singleton) or ORCA1622 and ORCA3835 (group 3) samples (Fig. 3B). However, analysis of DR_R_-T1 and the other repeats showed that the 42 ciHHV-6B carriers from Orkney fell into two groups that share the same length at DR_R_-T1 either with ORCA1340 or with ORCA1622 and ORCA3835 (Table 3). For junction fragment analysis, we selected ORCA1006 as a substitute for ORCA1340, since it shares the same DR_R_-T1 length. Similarly, ORCA1043, ORCA2119, and ORCA1263 were used as substitutes for ORCA1622 and ORCA3835, since they share a different DR_R_-TI length. Using the chromosome 17p primers, junction fragments were generated from all of the group 3 ciHHV-6B samples and from 1-ciHHV-6B (a singleton in the phylogenetic network) (Fig. 3). Using these primers, PCR products were not amplified from other ciHHV-6B samples in this study. The sequences of seven junction fragments from group 3 ciHHV-6B genomes (including NWA008 [36], which is another ciHHV-6B carrier with a viral genome that belongs to group 3 [data not shown]) were similar to each other but different from the fragment in sample 1-ciHHV-6B (Fig. 6B). These data indicate the existence of at least two independent integration events into different alleles of the chromosome 17p telomere, or possibly into telomeres of different chromosomes that have similar subterminal sequences (37).

Comparison of the junction fragments from group 3 ciHHV-6B samples showed remarkably similar TTAGGG and degenerate-repeat interspersion patterns (Fig. 6B). The differences among the interspersion patterns are consistent with small gains or losses that may have arisen from replication errors in the germ line after integration of the viral genome (32). Therefore, it is most likely that the ciHHV-6B status of group 3 individuals arose from a single ancestral integration event. Using the levels of nucleotide substitution between the group 3 ciHHV-6B genomes, the time to the most recent common ancestor (TMRCA) was estimated as 24,538 ± 10,625 years ago (Table 4). This estimate is based on the assumption that, once integrated, the ciHHV-6B genome mutates at the same average rate as the human genome as a whole. However, deviation from this rate would result in an under- or overestimation of the TMRCA.

**TABLE 4:** Estimate of TMRCA for ciHHV-6B genomes in group 3

Parameter	Value		
	Entire group 3^*a*^	HGDP1065 and HGDP1077	HGDP1065 and DER512
TMRCA (yrs ago)	24,538	23,004	15,336
Standard deviation	10,625	13,281	10,844

a ORCA1622 and ORCA3835 are identical across nonrepeat regions.

### Genetic intactness of ciHHV-6 genomes.

**FIG 7:**
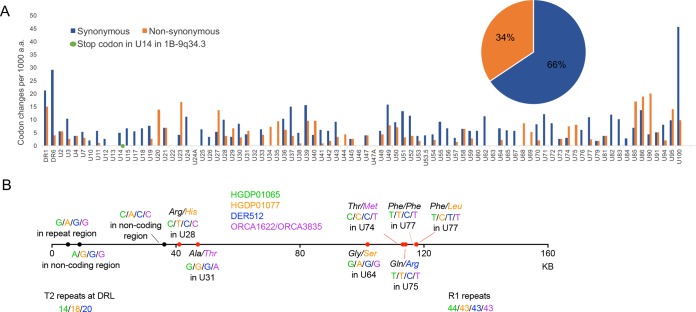
Consequences of nucleotide substitutions across the ciHHV-6 genome. (A) Comparison of synonymous (blue) and nonsynonymous (orange) substitution frequencies in each ciHHV-6B gene among the 21 ciHHV-6B genomes (scaled to differences per 1,000 amino acids). The green dot shows the novel in-frame stop codon in U14 of 1-ciHHV-6B. The pie chart shows the overall proportions of synonymous and nonsynonymous substitutions across all the genes. (B) Diagram showing the approximate locations and consequences of nucleotide substitutions that are predicted to have arisen after integration in group 3 ciHHV-6B genomes. The horizontal line represents the HHV-6B genome; black dots, locations of noncoding base substitutions; red dots, base substitutions within HHV-6B genes that are predicted to result in amino acid substitutions (nonsynonymous), as indicated; pink dot, synonymous (T-to-C) substitution in DER512 that is not predicted to change the phenylalanine. The numbers of repeats in three regions (T2, R1, and R4) that vary among the group 3 genomes are also shown.

The evidence for an ancient origin of some, probably most, of the ciHHV-6B genomes analyzed, and for postintegration mutations in repeat regions, raised the question of whether these genomes contain an intact set of viral genes or whether they have been rendered nonfunctional by mutation. To explore the consequences of sequence variation among the ciHHV6B genomes, the amino acid sequences predicted from all the genes in the ciHHV-6B genomes were aligned, and the cumulative frequencies of independent synonymous and nonsynonymous substitutions were determined (Fig. 7A). The ratio of synonymous to nonsynonymous substitutions varies among genes. The great majority of nonsynonymous changes (amounting to 34% of the total) result in single amino acid substitutions, but one substitution in the U20 stop codon of HGDP00092 is predicted to extend the coding region by eight codons. Only one substitution, which creates an in-frame stop codon in U14 of 1-ciHHV-6B, is predicted to terminate a coding region prematurely. Two of the seven ciHHV-6A genomes also have in-frame stop codons, one in U79 of GLA_15137 and the other in U83 genes of GLA_4298 (data not shown).

The 21 inherited ciHHV-6B genomes are likely to include mutations that arose before integration and represent variation among the parental nonintegrated HHV-6B strains, as well as mutations that arose after integration. To explore the latter, five group 3 ciHHV-6B genomes were compared (Fig. 7B). Among the 10 substitutions identified, 3 were in noncoding regions, 1 was a synonymous mutation in U77, and 6 were nonsynonymous mutations. From these limited data, it seems likely that the accumulation of mutations after integration has been random in these ciHHV-6B genomes.

## DISCUSSION

In this study, we used comparative analyses to explore diversity among ciHHV-6 genomes in order to understand the factors that influence the population frequencies of ciHHV-6 and to determine whether the integrated genomes appear to retain the capacity for full functionality as a virus. We found that the ciHHV-6B genomes are more similar to each another than to the two available HHV-6B reference genomes from Japan and the Democratic Republic of the Congo (Fig. 2, 3, and 4; see Fig. S1 in the supplemental material). This is particularly noticeable among the 19 ciHHV-6B genomes from individuals with European ancestry, which are more similar to each other than they are to the ciHHV-6B genomes in HGDP00092 from Pakistan and HGDP00813 from China. This pointer toward a relationship between the integrated HHV-6B strain and geographical distribution warrants further investigation if the association between carrier status and potential disease risk is to be understood fully (21, 38). The smaller group of seven ciHHV-6A genomes show higher levels of divergence from the three available HHV-6A reference genomes from the United States, Uganda, and the Gambia and as reported previously (30). However, in making these observations, the possibility of sample bias should be considered, both in the geographic distribution of the ciHHV-6 genomes analyzed and, in particular, in the small number of nonintegrated HHV-6A and HHV-6B genomes that are available for comparative analysis.

The isolation of chromosome junction fragments from eight ciHHV-6B samples (seven group 3 samples and 1-ciHHV-6B) by using primers from chromosome 17p subterminal sequences (35) suggests integration in alleles of the 17p telomere. Given the variable nature of human subterminal regions (37), the chromosome locations should be confirmed using a different approach. Nevertheless, comparison of the TTAGGG and degenerate-repeat interspersion patterns at the chromosome–ciHHV-6B junction can be used to deduce relationships (34, 39) and, combined with the phylogenetic analyses, shows that the individuals carrying a group 3 ciHHV-6B genome share an ancient ancestor. Group 3 includes individuals from Sardinia, England, Wales, and Orkney, with greater divergence between the ciHHV-6B genomes in the two individuals from Sardinia (HGDP1065 and HGDP1077) than between the individual from Derby, England (DER512), and the Sardinian (HGDP1065) (Fig. 3, 5, and 6 and Tables 1 and 3). Moreover, there is no evidence of a close family relationship between the two individuals from Sardinia. Overall, the data are consistent with the group 3 ciHHV-6B carriers being descendants of a common ancestor who existed approximately 24,500 years ago, similar to the date of the last glacial maximum and probably predating the colonization of Sardinia and Orkney.

The population screen of Orkney identified 42 ciHHV-6B carriers (frequency, 1.9%) (Table 2) and no ciHHV-6A carriers, which also suggests a founder effect. However, the Orkney ciHHV-6B samples can be divided into two groups based on the length of DR_R_-T1, the ciHHV-6B phylogenetic analyses, and the different integration sites. Therefore, it is likely that the ciHHV-6B carriers in Orkney are the descendants of two different ciHHV-6B ancestors who may have migrated to Orkney independently. This is consistent with the fine-resolution genetic structure of the Orkney population and the history of Orkney, which includes recent admixture from Norway (Norse Vikings) (36).

Given the evidence that extant ciHHV-6B carriers in group 3 are descendants of a single ancient founder with a germ line integration, it is plausible that other clusters in the phylogenetic tree have similar histories. For example, the three individuals in group 2 may all carry a ciHHV-6B genome integrated in a chromosome 11p telomere. Further verification is required to support this speculation, and this will be valuable when assessing disease risk associated with ciHHV-6 integrations in different telomeres.

There is good evidence that ciHHV-6 genomes can reactivate in some settings, for example, when the immune system is compromised (19, 20). However, it is not known what proportion of ciHHV-6 genomes may retain the capacity to reactivate. We investigated this question from various angles. We have presented evidence that some ciHHV-6 genomes are ancient and therefore could have accumulated inactivating mutations while in the human genome. Most of the tandem repeats analyzed in ciHHV-6B genomes showed minor variations in repeat copy numbers (Fig. 5 and Table 3). However, the functions of these regions are unclear, and as copy number variation exists among the reference genomes, it seems unlikely that the level of variation detected unduly influences the potential functionality of the integrated viral genomes. In the protein-coding regions of ciHHV-6B genomes, 34% of substitutions are nonsynonymous and are predicted to cause amino acid substitutions (Fig. 7). A single potentially inactivating mutation was detected as an in-frame stop codon in gene U14 in 1-ciHHV-6B. Since this gene encodes a tegument protein that is essential for the production of viral particles and can induce cell cycle arrest at the G_2_/M phase (40), it seems unlikely that this integrated copy of ciHHV-6B would be able to reactivate. However, the other viral genes may be expressed in this ciHHV-6B genome, and the presence of the viral genome may also affect telomere function. The stop codon in gene U20 in the individual from Pakistan (HGDP00092) is mutated, and this is predicted to extend the U20 protein by 8 amino acid residues. U20 is part of a cluster of genes (U20 to U24) that are specific to HHV-6A, HHV-6B, and their relative human betaherpesvirus 7 and likely plays a role in suppressing an apoptotic response by the infected host cell (41, 42). Further experimental analysis will be required to determine whether the modest extension affects the function of the U20 protein. Among the seven ciHHV-6A genomes, two contain novel in-frame stop codons. One of these is located in U83 in GLA_4298. The other is present in U79 in GLA_15137, but this inactivating mutation is absent from the closely related ciHHV-6A genome in 7A-17p13.3 (Fig. 3C and D).

In summary, we have shown that most ciHHV-6A and ciHHV-6B genomes contain an intact set of genes and therefore may have the potential to be fully functional. This observation needs to be taken into consideration when assessing whether ciHHV-6 carrier status is associated with disease risk and in understanding the underlying mechanisms of such associations (e.g., whether viral reactivation is involved). Among the individuals of European descent, we found strong evidence for the ancient common ancestry of some of the integrated viral genomes. The close similarity between ciHHV-6B genomes in the Europeans and the evidence of multiple different integration events by similar strains also indicate that we have effectively sequenced the ancient, nonintegrated strains of HHV-6B that existed in European populations in prehistoric times. Based on these observations, it is possible that other populations, for example, in China, South Asia, and Africa, may show similar founder effects among ciHHV-6 carriers but from different ancient strains (43). Our limited knowledge of nonintegrated HHV-6A and HHV-6B strains is based mostly on strains derived from Africa and Japan. There is now a real need to sequence nonintegrated strains from other populations, including those in Europe, so that the relationship between nonintegrated HHV-6 and ciHHV-6 can be fully understood. A major challenge will be to determine whether germ line integration continues to occur *de novo* today and, if so, at what rate and by which viral strains.

## MATERIALS AND METHODS

### Population screening to identify ciHHV-6 carriers.

ciHHV-6 carriers were identified by screening a variety of DNA sample collections from individuals from around the world, using PCR assays to detect either U11, U18, DR5 (HHV-6A), or DR7 (HHV-6B) (12), or U7, DR1, DR6A, or DR6B (22 and unpublished data). DR5, DR6A, DR6B, and DR7 correspond to ORFs in the original annotation of the HHV-6A genome (GenBank accession no. X83413) (25), but DR5 is in a noncoding region of the genome, and DR6A, DR6B, and DR7 are in exons of DR6 in the reannotation used (RefSeq accession no. NC_001664). From the populations screened, 58 samples with ciHHV-6 among 3,875 individuals were identified (Table 2). The number of individuals screened in most populations was small and therefore cannot be used to give an accurate estimate of ciHHV-6A or -B frequencies, although a larger number of ciHHV-6B-positive samples were identified overall. The frequency of ciHHV-6B carriers in Orkney (1.9%), a collection of islands off the north coast of Scotland, is higher than that reported from England (44). Screening of the GS:SFHS will be described elsewhere (R. F. Jarrett, unpublished data). Ethical approval for the GS:SFHS cohort was obtained from the Tayside Committee on Medical Research Ethics (on behalf of the National Health Service).

### Generation of overlapping amplicons and sequencing.

The 32 primer pairs used to generate overlapping amplicons from ciHHV-6A genomes and the PCR conditions employed were reported previously (18). The primer pairs used to amplify ciHHV-6B genomes were based on conserved sequences from the HHV-6B nonintegrated HST and Z29 strains (GenBank accession no. AB021506.1 and AF157706, respectively) (9, 24). The primer sequences are shown in Table S1 in the supplemental material. The amplicons from each sample were pooled in equimolar proportions and then sequenced using the Illumina MiSeq or IonTorrent (Life Technologies) next-generation sequencing platforms, as described previously (18). Some sequences were verified by using Sanger dideoxy chain termination sequencing on PCR-amplified products.

### Assembly and analysis of DNA sequence data.

DNA sequence data were processed essentially as described previously (18), except that SPAdes v. 3.5.0 (45) was used for *de novo* assembly into contigs, ABACAS v. 1.3.1 (46) was used to order contigs, and Gapfiller v. 1-11 (47) was used to fill gaps between contigs. The integrity of the sequences was verified by aligning them against the read data using BWA v. 0.6.2-r126 (53) and visualizing the alignments as BAM files using Tablet v. 1.13.08.05 (52). Nucleotide substitutions, indels, and repeat regions were also verified by manual analysis using IGV v. 2.3 (http://software.broadinstitute.org/software/igv/home).

Alignments of the seven ciHHV-6A genomes with the three published HHV-6A genomes from the nonintegrated strains U1102, GS, and AJ (25, 27–29) and alignment of the 21 ciHHV-6B genomes with the two previously published HHV-6B genomes from the nonintegrated viruses HST and Z29 (9, 24) were carried out using Gap4 (48). Variation across the ciHHV-6 genomes was studied by a combination of manual inspection and automated analysis using an in-house Perl script. The script performed a sliding-window count of substitutions using the aligned Gap4 files, reporting the count according to the midpoint of the window. For analysis across the genome, the window size was 1 kb and the step size was 1 nucleotide. For analysis of individual ORFs, a file with a list of annotated positions was generated.

Phylogenetic analyses were carried out by two different methods. Maximum-likelihood trees were built by using the maximum composite likelihood model (MEGA6.0), and bootstrap values were obtained with 2,000 replications. Model selection was carried out for HHV-6A and HHV-6B separately, and the substitution model with the lowest Bayesian information criterion was selected (the Tamura 3-parameter model [49] for HHV-6B and the Hasegawa-Kishino-Yano model for HHV-6A). Median-joining networks were built using Network 5.0 (Fluxus Engineering) with default parameters. Sites with missing data were excluded from all phylogenetic analyses for both HHV-6A and HHV-6B. The number of positions analyzed for HHV-6B was 130,412, and that for HHV-6A was 117,900. The TMRCA was calculated by using rho as implemented in Network 5.0. Rho values were transformed into time values using the accepted mutation rate for the human genome, 0.5E−9 substitutions per bp per year (50), scaled to the number of sites analyzed.

### Comparison of tandem-repeat regions.

The copy numbers of repeat units in the DR-R, R0, R1, R2, R3, and R4 tandem-repeat regions (9, 24) were determined by manual inspection of the individual BAM files generated for each sequenced ciHHV-6 genome, with verification by checking the sequence alignments generated using Gap4. The number of copies of TTAGGG in each DR_L_-T2 region was determined from PCR amplicons generated using the DR8F and UDL6R primers (see Table S1 in the supplemental material). Each amplicon was purified using a Zymoclean gel DNA recovery kit (Cambridge Bioscience) and then sequenced by the Sanger dideoxy chain termination method. The sequence data were analyzed by using MacVector software (MacVector Inc.). Variation at the (CA)_*n*_ repeat array located immediately adjacent to T1 in HHV-6B was investigated in DR_L_ specifically by reamplification of STELA (51) products, using the primers DR1R and TJ1F. The short amplicons were purified and sequenced as described above and compared with the same sequences in the reference HST and Z29 genomes.

### Analysis of the DR_R_-T1 region by TVR-PCR.

The DR_R_-T1 regions from ciHHV-6B-positive samples were amplified using the primers U100Fw2 and DR1R. TVR-PCR was conducted on each of these amplicons essentially as described previously (32, 33) but using an end-labeled primer, HHV-6B-UDR5F, and the unlabeled TAG-TELWRev. The TELWRev primer anneals to TTAGGG repeats, allowing amplification of products that differ in length depending on the location of the TTAGGG repeat with respect to the flanking primer (HHV-6B-UDR5F). The labeled amplicons from the T1 region were separated by size in a 6% denaturing polyacrylamide gel.

### Analysis of HHV-6 ORFs.

The frequency of nucleotide substitutions in each ORF was determined by a combination of manual inspection and automated analysis using a Perl script, as described above. The DNA sequences of the 86 HHV-6B ORFs from the 21 ciHHV-6B genomes were aligned to identify and compare the numbers of synonymous and nonsynonymous codon changes within and among genes. In addition, the predicted amino acid sequences for each gene in the 21 ciHHV-6B genomes were aligned to confirm the number of nonsynonymous changes.

### Characterization of chromosome-ciHHV-6 junctions.

The junctions between the chromosome and the ciHHV-6 genome were isolated by PCR amplification using various primers that anneal to subterminal regions of a variety of human chromosomes in combination with the DR8F primer. The amplicons were purified as described above and sequenced by the Sanger method with a variety of primers (see Table S1 in the supplemental material). The number of repeats present in each junction fragment and the interspersion of TTAGGG repeats with degenerate repeats was determined by manual inspection using MacVector software.

### Accession number(s).

The finished sequences have been deposited in GenBank under accession numbers KY316030 to KY316056 (Table 1). The LEI_1501 ciHHV-6A genome reported previously has accession number KT355575 (18).

## Supplementary Material

Supplemental material

## References

[1] Holohan B, Wright WE, Shay JW 2014 Cell biology of disease: telomeropathies: an emerging spectrum disorder. J Cell Biol 205:289–299. doi:10.1083/jcb.201401012. 24821837 PMC4018777

[2] Reddel RR 2010 Senescence: an antiviral defense that is tumor suppressive? Carcinogenesis 31:19–26. doi:10.1093/carcin/bgp274. 19887513

[3] Ablashi D, Agut H, Alvarez-Lafuente R, Clark DA, Dewhurst S, DiLuca D, Flamand L, Frenkel N, Gallo R, Gompels UA, Hollsberg P, Jacobson S, Luppi M, Lusso P, Malnati M, Medveczky P, Mori Y, Pellett PE, Pritchett JC, Yamanishi K, Yoshikawa T 2014 Classification of HHV-6A and HHV-6B as distinct viruses. Arch Virol 159:863–870. doi:10.1007/s00705-013-1902-5. 24193951 PMC4750402

[4] de Lange T 2005 Shelterin: the protein complex that shapes and safeguards human telomeres. Genes Dev 19:2100–2110. doi:10.1101/gad.1346005. 16166375

[5] Sfeir A, de Lange T 2012 Removal of shelterin reveals the telomere end-protection problem. Science 336:593–597. doi:10.1126/science.1218498. 22556254 PMC3477646

[6] Arnoult N, Karlseder J 2015 Complex interactions between the DNA-damage response and mammalian telomeres. Nat Struct Mol Biol 22:859–866. doi:10.1038/nsmb.3092. 26581520 PMC4739752

[7] Lindquester GJ, Pellett PE 1991 Properties of the human herpesvirus 6 strain Z29 genome: G+C content, length, and presence of variable-length directly repeated terminal sequence elements. Virology 182:102–110. doi:10.1016/0042-6822(91)90653-S. 2024458

[8] Achour A, Malet I, Deback C, Bonnafous P, Boutolleau D, Gautheret-Dejean A, Agut H 2009 Length variability of telomeric repeat sequences of human herpesvirus 6 DNA. J Virol Methods 159:127–130. doi:10.1016/j.jviromet.2009.03.002. 19442857

[9] Dominguez G, Dambaugh TR, Stamey FR, Dewhurst S, Inoue N, Pellett PE 1999 Human herpesvirus 6B genome sequence: coding content and comparison with human herpesvirus 6A. J Virol 73:8040–8052. 10482553 10.1128/jvi.73.10.8040-8052.1999 PMC112820

[10] De Bolle L, Naesens L, De Clercq E 2005 Update on human herpesvirus 6 biology, clinical features, and therapy. Clin Microbiol Rev 18:217–245. doi:10.1128/CMR.18.1.217-245.2005. 15653828 PMC544175

[11] Arbuckle JH, Medveczky MM, Luka J, Hadley SH, Luegmayr A, Ablashi D, Lund TC, Tolar J, De Meirleir K, Montoya JG, Komaroff AL, Ambros PF, Medveczky PG 2010 The latent human herpesvirus-6A genome specifically integrates in telomeres of human chromosomes in vivo and in vitro. Proc Natl Acad Sci U S A 107:5563–5568. doi:10.1073/pnas.0913586107. 20212114 PMC2851814

[12] Huang Y, Hidalgo-Bravo A, Zhang E, Cotton VE, Mendez-Bermudez A, Wig G, Medina-Calzada Z, Neumann R, Jeffreys AJ, Winney B, Wilson JF, Clark DA, Dyer MJ, Royle NJ 2014 Human telomeres that carry an integrated copy of human herpesvirus 6 are often short and unstable, facilitating release of the viral genome from the chromosome. Nucleic Acids Res 42:315–327. doi:10.1093/nar/gkt840. 24057213 PMC3874159

[13] Daibata M, Taguchi T, Nemoto Y, Taguchi H, Miyoshi I 1999 Inheritance of chromosomally integrated human herpesvirus 6 DNA. Blood 94:1545–1549. 10477678

[14] Morris C, Luppi M, McDonald M, Barozzi P, Torelli G 1999 Fine mapping of an apparently targeted latent human herpesvirus type 6 integration site in chromosome band 17p13.3. J Med Virol 58:69–75. doi:10.1002/(SICI)1096-9071(199905)58:1<69::AID-JMV11>3.0.CO;2-3. 10223549

[15] Tanaka-Taya K, Sashihara J, Kurahashi H, Amo K, Miyagawa H, Kondo K, Okada S, Yamanishi K 2004 Human herpesvirus 6 (HHV-6) is transmitted from parent to child in an integrated form and characterization of cases with chromosomally integrated HHV-6 DNA. J Med Virol 73:465–473. doi:10.1002/jmv.20113. 15170644

[16] Nacheva EP, Ward KN, Brazma D, Virgili A, Howard J, Leong HN, Clark DA 2008 Human herpesvirus 6 integrates within telomeric regions as evidenced by five different chromosomal sites. J Med Virol 80:1952–1958. doi:10.1002/jmv.21299. 18814270

[17] Prusty BK, Krohne G, Rudel T 2013 Reactivation of chromosomally integrated human herpesvirus-6 by telomeric circle formation. PLoS Genet 9:e1004033. doi:10.1371/journal.pgen.1004033. 24367281 PMC3868596

[18] Zhang E, Cotton VE, Hidalgo-Bravo A, Huang Y, Bell JA, Jarrett FR, Wilkie GS, Davison AJ, Nacheva PE, Siebert R, Majid A, Kelpanides I, Jayne S, Dyer MJ, Royle NJ 2016 HHV-8-unrelated primary effusion-like lymphoma associated with clonal loss of inherited chromosomally-integrated human herpesvirus-6A from the telomere of chromosome 19q. Sci Rep 6:22730. doi:10.1038/srep22730. 26947392 PMC4779988

[19] Endo A, Watanabe K, Ohye T, Suzuki K, Matsubara T, Shimizu N, Kurahashi H, Yoshikawa T, Katano H, Inoue N, Imai K, Takagi M, Morio T, Mizutani S 2014 Molecular and virological evidence of viral activation from chromosomally integrated human herpesvirus 6A in a patient with X-linked severe combined immunodeficiency. Clin Infect Dis 59:545–548. doi:10.1093/cid/ciu323. 24803376

[20] Gravel A, Hall CB, Flamand L 2013 Sequence analysis of transplacentally acquired human herpesvirus 6 DNA is consistent with transmission of a chromosomally integrated reactivated virus. J Infect Dis 207:1585–1589. doi:10.1093/infdis/jit060. 23408849

[21] Gravel A, Dubuc I, Morissette G, Sedlak RH, Jerome KR, Flamand L 2015 Inherited chromosomally integrated human herpesvirus 6 as a predisposing risk factor for the development of angina pectoris. Proc Natl Acad Sci U S A 112:8058–8063. doi:10.1073/pnas.1502741112. 26080419 PMC4491735

[22] Bell AJ, Gallagher A, Mottram T, Lake A, Kane EV, Lightfoot T, Roman E, Jarrett RF 2014 Germ-line transmitted, chromosomally integrated HHV-6 and classical Hodgkin lymphoma. PLoS One 9:e112642. doi:10.1371/journal.pone.0112642. 25384040 PMC4226568

[23] Moustafa A, Xie C, Kirkness E, Biggs W, Wong E, Turpaz Y, Bloom K, Delwart E, Nelson KE, Venter JC, Telenti A 2017 The blood DNA virome in 8,000 humans. PLoS Pathog 13:e1006292. doi:10.1371/journal.ppat.1006292. 28328962 PMC5378407

[24] Isegawa Y, Mukai T, Nakano K, Kagawa M, Chen J, Mori Y, Sunagawa T, Kawanishi K, Sashihara J, Hata A, Zou P, Kosuge H, Yamanishi K 1999 Comparison of the complete DNA sequences of human herpesvirus 6 variants A and B. J Virol 73:8053–8063. 10482554 10.1128/jvi.73.10.8053-8063.1999 PMC112821

[25] Gompels UA, Nicholas J, Lawrence G, Jones M, Thomson BJ, Martin ME, Efstathiou S, Craxton M, Macaulay HA 1995 The DNA sequence of human herpesvirus-6: structure, coding content, and genome evolution. Virology 209:29–51. doi:10.1006/viro.1995.1228. 7747482

[26] Downing RG, Sewankambo N, Serwadda D, Honess R, Crawford D, Jarrett R, Griffin BE 1987 Isolation of human lymphotropic herpesviruses from Uganda. Lancet ii:390. 10.1016/s0140-6736(87)92403-2 2886840

[27] Salahuddin SZ, Ablashi DV, Markham PD, Josephs SF, Sturzenegger S, Kaplan M, Halligan G, Biberfeld P, Wong-Staal F, Kramarsky B, Gallo R 1986 Isolation of a new virus, HBLV, in patients with lymphoproliferative disorders. Science 234:596–601. doi:10.1126/science.2876520. 2876520

[28] Gravel A, Ablashi D, Flamand L 2013 Complete genome sequence of early passaged human herpesvirus 6A (GS strain) isolated from North America. Genome Announc 1:e00012-13. doi:10.1128/genomeA.00012-13. 23766398 PMC3707569

[29] Tweedy J, Spyrou MA, Donaldson CD, Depledge D, Breuer J, Gompels UA 2015 Complete genome sequence of the human herpesvirus 6A strain AJ from Africa resembles strain GS from North America. Genome Announc 3:e01498-14. doi:10.1128/genomeA.01498-14. 25676750 PMC4333650

[30] Tweedy J, Spyrou MA, Pearson M, Lassner D, Kuhl U, Gompels UA 2016 Complete genome sequence of germline chromosomally integrated human herpesvirus 6A and analyses integration sites define a new human endogenous virus with potential to reactivate as an emerging infection. Viruses 8:E19. doi:10.3390/v8010019. 26784220 PMC4728579

[31] Chakraborty R, Kimmel M, Stivers DN, Davison LJ, Deka R 1997 Relative mutation rates at di-, tri-, and tetranucleotide microsatellite loci. Proc Natl Acad Sci U S A 94:1041–1046. doi:10.1073/pnas.94.3.1041. 9023379 PMC19636

[32] Baird DM, Jeffreys AJ, Royle NJ 1995 Mechanisms underlying telomere repeat turnover, revealed by hypervariable variant repeat distribution patterns in the human Xp/Yp telomere. EMBO J 14:5433–5443. 7489732 10.1002/j.1460-2075.1995.tb00227.x PMC394652

[33] Varley H, Pickett HA, Foxon JL, Reddel RR, Royle NJ 2002 Molecular characterization of inter-telomere and intra-telomere mutations in human ALT cells. Nat Genet 30:301–305. doi:10.1038/ng834. 11919561

[34] Mendez-Bermudez A, Hills M, Pickett HA, Phan AT, Mergny JL, Riou JF, Royle NJ 2009 Human telomeres that contain (CTAGGG)n repeats show replication dependent instability in somatic cells and the male germline. Nucleic Acids Res 37:6225–6238. doi:10.1093/nar/gkp629. 19656953 PMC2764434

[35] Britt-Compton B, Rowson J, Locke M, Mackenzie I, Kipling D, Baird DM 2006 Structural stability and chromosome-specific telomere length is governed by cis-acting determinants in humans. Hum Mol Genet 15:725–733. doi:10.1093/hmg/ddi486. 16421168

[36] Leslie S, Winney B, Hellenthal G, Davison D, Boumertit A, Day T, Hutnik K, Royrvik EC, Cunliffe B, Wellcome Trust Case Control Consortium 2, International Multiple Sclerosis Genetics Consortium, Lawson DJ, Falush D, Freeman C, Pirinen M, Myers S, Robinson M, Donnelly P, Bodmer W 2015 The fine-scale genetic structure of the British population. Nature 519:309–314. doi:10.1038/nature14230. 25788095 PMC4632200

[37] Riethman H 2008 Human telomere structure and biology. Annu Rev Genomics Hum Genet 9:1–19. doi:10.1146/annurev.genom.8.021506.172017. 18466090

[38] Pinto EM, Chen X, Easton J, Finkelstein D, Liu Z, Pounds S, Rodriguez-Galindo C, Lund TC, Mardis ER, Wilson RK, Boggs K, Yergeau D, Cheng J, Mulder HL, Manne J, Jenkins J, Mastellaro MJ, Figueiredo BC, Dyer MA, Pappo A, Zhang J, Downing JR, Ribeiro RC, Zambetti GP 2015 Genomic landscape of paediatric adrenocortical tumours. Nat Commun 6:6302. doi:10.1038/ncomms7302. 25743702 PMC4352712

[39] Mendez-Bermudez A, Hidalgo-Bravo A, Cotton VE, Gravani A, Jeyapalan JN, Royle NJ 2012 The roles of WRN and BLM RecQ helicases in the alternative lengthening of telomeres. Nucleic Acids Res 40:10809–10820. doi:10.1093/nar/gks862. 22989712 PMC3510502

[40] Mori J, Kawabata A, Tang H, Tadagaki K, Mizuguchi H, Kuroda K, Mori Y 2015 Human herpesvirus-6 U14 induces cell-cycle arrest in G_2_/M phase by associating with a cellular protein, EDD. PLoS One 10:e0137420. doi:10.1371/journal.pone.0137420. 26340541 PMC4560387

[41] Kofod-Olsen E, Ross-Hansen K, Schleimann MH, Jensen DK, Moller JM, Bundgaard B, Mikkelsen JG, Hollsberg P 2012 U20 is responsible for human herpesvirus 6B inhibition of tumor necrosis factor receptor-dependent signaling and apoptosis. J Virol 86:11483–11492. doi:10.1128/JVI.00847-12. 22896603 PMC3486335

[42] Jasirwan C, Tang H, Kawabata A, Mori Y 2015 The human herpesvirus 6 U21-U24 gene cluster is dispensable for virus growth. Microbiol Immunol 59:48–53. doi:10.1111/1348-0421.12208. 25346365

[43] Kawamura Y, Ohye T, Miura H, Ihira M, Kato Y, Kurahashi H, Yoshikawa T 2017 Analysis of the origin of inherited chromosomally integrated human herpesvirus 6 in the Japanese population. J Gen Virol 98:1823–1830. doi:10.1099/jgv.0.000834. 28699856

[44] Leong HN, Tuke PW, Tedder RS, Khanom AB, Eglin RP, Atkinson CE, Ward KN, Griffiths PD, Clark DA 2007 The prevalence of chromosomally integrated human herpesvirus 6 genomes in the blood of UK blood donors. J Med Virol 79:45–51. doi:10.1002/jmv.20760. 17133548

[45] Bankevich A, Nurk S, Antipov D, Gurevich AA, Dvorkin M, Kulikov AS, Lesin VM, Nikolenko SI, Pham S, Prjibelski AD, Pyshkin AV, Sirotkin AV, Vyahhi N, Tesler G, Alekseyev MA, Pevzner PA 2012 SPAdes: a new genome assembly algorithm and its applications to single-cell sequencing. J Comput Biol 19:455–477. doi:10.1089/cmb.2012.0021. 22506599 PMC3342519

[46] Assefa S, Keane TM, Otto TD, Newbold C, Berriman M 2009 ABACAS: algorithm-based automatic contiguation of assembled sequences. Bioinformatics 25:1968–1969. doi:10.1093/bioinformatics/btp347. 19497936 PMC2712343

[47] Boetzer M, Pirovano W 2012 Toward almost closed genomes with GapFiller. Genome Biol 13:R56. doi:10.1186/gb-2012-13-6-r56. 22731987 PMC3446322

[48] Staden R, Beal KF, Bonfield JK 2000 The Staden package, 1998. Methods Mol Biol 132:115–130. 10547834 10.1385/1-59259-192-2:115

[49] Tamura K, Stecher G, Peterson D, Filipski A, Kumar S 2013 MEGA6: Molecular Evolutionary Genetics Analysis version 6.0. Mol Biol Evol 30:2725–2729. doi:10.1093/molbev/mst197. 24132122 PMC3840312

[50] Scally A, Durbin R 2012 Revising the human mutation rate: implications for understanding human evolution. Nat Rev Genet 13:745–753. doi:10.1038/nrg3295. 22965354

[51] Baird DM, Rowson J, Wynford-Thomas D, Kipling D 2003 Extensive allelic variation and ultrashort telomeres in senescent human cells. Nat Genet 33:203–207. doi:10.1038/ng1084. 12539050

[52] Milne I, Stephen G, Bayer M, Cock PJ, Pritchard L, Cardle L, Shaw PD, Marshall D 2013 Using Tablet for visual exploration of second-generation sequencing data. Brief Bioinform 14:193–202. doi:10.1093/bib/bbs012. 22445902

[53] Li H, Durbin R 2009 Fast and accurate short read alignment with Burrows-Wheeler transform. Bioinformatics 25:1754–1760. doi:10.1093/bioinformatics/btp324. 19451168 PMC2705234

