# Supplementary material for: Inherited Chromosomally Integrated Human Herpesvirus 6 Genomes Are Ancient, Intact, and Potentially Able To Reactivate from Telomeres
Source: J Virol. 2017 Oct 27;91(22):e01137-17. doi: 10.1128/JVI.01137-17 (PMC5660504; doi:10.1128/JVI.01137-17)
Supplement: Supplemental material [file supp_91_22_e01137-17__index.html]

Inherited Chromosomally Integrated Human Herpesvirus 6 Genomes Are Ancient, Intact, and Potentially Able To Reactivate from Telomeres — Supplemental material 

# Inherited Chromosomally Integrated Human Herpesvirus 6 Genomes Are Ancient, Intact, and Potentially Able To Reactivate from Telomeres

## Supplemental material

- Supplemental file 1 -

  Table S1 (Primers for HHV-6 detection, identification, and characterization.)

  Fig. S1 (Phylogenetic analysis of HHV-6 DR regions.)

  Fig. S2 (Maximum-likelihood phylogenetic trees of individual genes from 21 inherited ciHHV-6B and 2 HHV-6B reference genomes (HST and Z29).)

  Fig. S3 (Maximum-likelihood phylogenetic trees of individual genes from seven inherited ciHHV-6A and three HHV-6A reference genomes (U1102, AJ, and GS).)

  PDF, 366K
