# Supplementary material for: Inherited Chromosomally Integrated Human Herpesvirus 6 Genomes Are Ancient, Intact, and Potentially Able To Reactivate from Telomeres
Source: J Virol. 2017 Oct 27;91(22):e01137-17. doi: 10.1128/JVI.01137-17 (PMC5660504; doi:10.1128/JVI.01137-17)

Zhang et al. Supplementary Tables and Figures

Table S1. Primers for HHV-6 detection, identification and characterization.

Primers for HHV-6B overlapping amplicons	Primer sequence	Primer position in HHV-6B, strain HST (Acc. No: AB021506)	
DR1F (HST/KUK)	ACCTTGGCCCGAGCAAGAATGC	638	658
DR8RB	CGCCCGCGACTGCCATAGAG	8168	8149
Pac2F	TGGGAGGCGCCGTGTTTTTC	8480	8499
HHV6 probe 10 R	TTGCATCGATACCGTTTCTG	12828	12809
Primer A1	TCACCGGATTCGACATGTAA	12518	12537
Probe A2	TTAAAGTACGGGGTGCAAGG	18237	18218
HHV6 probe 14 F	ACACTCGCATTCCGAAAGTT	18157	18176
HHV6 probe 14 R	AGTCGCTGAGTCCTTGGGTA	19855	19836
HHV6 probe 5F (U11)	TTTTTACATCACGACGCGATC	19793	19813
HHV6 probe 5R	ATGGTCCTCCATGGGTTCTT	25767	25748
HHV6 probe 18 F	ATGGCGCACGCTAAAAAG	23699	23716
HHV6 probe 6R2	TGTAACGGAGGAATGGGAAG	31513	31494
HHV6 probe 16 F	CCAAGCACTTGACCAGTTGA	31430	31449
HHV6 probe 16 R	TTGTCGGGACTGATGATGAA	38065	38046
HHV6 probe 17 F	TCGAACTAAACCCGAGACCT	37990	38009
HHV6 probe 17 R	GCCATGTGGTTTGAGAGGAT	44442	44423
HHV6 probe 7F	CGAGAAGCTCATGCTTACCC	44319	44338
HHV6 probe 7R	AACTTGAAGCTGGCGACATT	50318	50299
HHV6 probe 13 F	TCTCACTTCCGAAACTTCTATGC	50223	50245
HHV6 probe 13 R	CAGTTGTTGCTTCCGATTCC	53624	53605
Probe B1	TCGGTCTGCTCATAGTCACG	53545	53564
Probe B2	CAGGGCTGTTGTCGGTAAAT	59130	59111
HHV6 probe 15 F	CGTGACGTGTGCCAATCT	59016	59033
HHV6 probe 15 R	GCTCAGTTGTCGAGGGAGAC	63122	63103
HHV6 probe 33 F	TCATCTTGCTCATTCTCCCTAA	63022	63043
HHV6 probe 33 R	TCACTTGCAGTCGTTTGAGG	68230	68211
HHV6 probe 32 F	ACACGCAACATGGCAAATAA	68122	68141
HHV6 probe 32 R	CGTGCCATAGCGAAATGTAA	73235	73216
HHV6 probe 31 F	TCGGAAGCGGAGATTTCTAA	73144	73163
HHV6 probe 31 R	CCACCTTCGCAACAACAATA	77701	77682
HHV6 probe 30 F	TGAATTTTGTCGTGCTCTGC	77591	77610
HHV6 probe 30 R	ATGCTTGCCTTTTCTCATGG	82740	82721
HHV6 probe 29 F	TGTCTCTCCTTCTGGCACCT	82659	82678
HHV6 probe 29 R	GCAATCTTAGCAGCCGACTC	86410	86391
HHV6 probe 23 F	AGCAGCCGAAAGTAGTTCCA	86317	86336
HHV6 probe 23 R	TCGGCGGAATTAACAAAAAC	91648	91629
Probe C1	CGCAGATAGCTTGTTGACCA	91589	91608
Probe C2	CACTTCAGTTCCAGGGGTGT	97721	97702
HHV6 probe 11 F	CGGAAACCATAGCTGTCCAT	97415	97434
HHV6 probe 11R	GCTTATGCTTCCCAATTCCA	102007	101988
HHV6 probe 24 F	GCGGTAAACGGCATACATTT	101897	101916
HHV6 probe 24 R	TGTACCTGGCAGCATCTGAG	104562	104543
HHV6 probe 25 F	ATTGTTTATGCGTGCAGACG	104510	104529
HHV6 probe 25 R	CCGTTGCTTTCTCTTCCATC	108762	108743
Probe D1	GGGTTTAACGTAGCGAACCA	108671	108690

Probe D2	CCGGAGAATGAAATCCTTGA	114565	114546
HHV6 probe 12 F	CATGGTTCCGTCTTCCAAGT	114379	114398
HHV6 probe 12 R	TGTGTGGAAACACCCTTCAA	120115	120096
HHV6 probe 19 F	CGGAGATATGACAAGTAGAGAGAGG	120053	120077
HHV6 probe 19 R	CAATGAATTCCTCAGCGACT	124330	124311
HHV6 probe 9F (U83 A/BF)	GCGCAAACAATGTGCGTAGT	124263	124282
HHV6 probe 9R	TCTCCTCTTCCGATGACACC	130529	130510
HHV6 probe 20 F	GTGTGGTGGAGTTCCGAGTT	130444	130463
HHV6 probe 20 R	TGATCCATTCGAAGAAAATGC	134841	134821
HHV6 probe 21 F	CACATCTGTATGCTAATGATTGCT	134756	134779
HHV6 probe 21 R	AGATTGATTGCACCCGAAAC	139157	139138
HHV6 probe 27 F	CAAGGTGGAGGTTTCTTTGG	139099	139118
HHV6 probe 27 R	AGGACCGTGTCCCATCATAG	142911	142892
HHV6 probe 28 R	TGGATATTTGAATGTACCATCGAG	142846	142869
HHV6 probe 28 R	TCGTGTTTAGAGTCCCGGTAA	147255	147235
HHV6 probe 26 F	CATGGTGGTTCTCCTGTGTG	147190	147209
HHV6 probe 26 R	GAGGGTGGGCACGTATTTTA	149441	149422
HHV6 probe 22 F	AACGGTCAGGTTCTCACGAC	149347	149366
HHV6 probe 22 R	TATCTGTCTTCCAGAGCAACAG	153042	153021
U100Fw2	TATCTCCGAACATGATGCTG	151384	151403
DR1R	GAAGAAGATGCGGTTGTCTTGTT	153849	153827
TJ1F	AACCCTAAGTCTAGCCCTTG		
**Primer -DR region**	**Primer sequence**	**Primer position in HST**	
U100Fw2	TATCTCCGAACATGATGCTG	151384	151403
DR1R	GAAGAAGATGCGGTTGTCTTGTT	153849	153827
DR8F	CATAGATCGGGACTGCTTGAA	7630	7650
UDL6R	TTTCGCTCACGTGGCAGTCT	9061	9042
HST5308F	CCAGATCCGTTCAAGACTGC	5308	5327
HST6376R	CGCAACATACGTACAGACTC	6395	6376
Pac2F	TGGGAGGCGCCGTGTTTTTC	8480	8499
U2-3R	GATCAGGTCGGCAGGGTGTA	9787	9768
HST9SeqF	AAAGGACTGGAGTCGAGCTG	128658	128677
HST9SeqR	CAAATCCAACAGTGGCAACA	129660	129641
HST139597F	GGAAATTAGCTGATGCTTAC	139597	139616
HST27Seq2R	AGGCGTGGTAGGAAATGATG	142503	142484
HST22SeqF	CGGGCCACTTCTTTTCATAC	152552	152571
Probe22R	TATCTGTCTTCCAGAGCAACAG	153042	153021
**Primers for internal junction**	**Primer sequence**	**Primer position in HST**	
SubT17-539	CCCAATTTACTGGTAATGGACT		
17p311	GAATCCACGGATTGCTTTGTGTAC		
DR8F	CATAGATCGGGACTGCTTGAA	7630/160672	7650/160692
HST161232F	GGGCAGATGTAAAGTCAATG	161232	161251
HST160957F	TATACGGAAGTGCATGCGAC	160957	160976
HST160892F	CATGACATTGAATACACGTGC	160892	160912
**Primers for TVR-PCR of HHV-6B -T1**	**Primer sequence**		
HHV-6B-UDR5F	CTGTTGCTCTGGAAGACAGAT		
TAG-TELWRev	TCATGCGTCCATGGTCCGGAGGGTAAGGGTAAGGGTNAGGGTT		

Primers for virus detection and identification	Primer sequence	Primer position in HST		Primer position in HHV-6A, strain U1102 (Acc. No. X83413)	
DR5F (A)	CACATACATGAACG GACACAC	-	-	3776/155009	3798/155031
DR5R (A)	CGTCGACTTCTCGT TCTTTATGC	-	-	4118/155351	4096/155329
DR6F (B)	AGGCGTGATTCTGG GAAAC	6952/159994	6970/160012	-	-
DR6R (B)	CCGAATACGTCCAA TGTCCT	7140/160182	7121/160163	-	-
DR7F	ACGAACACGACCTG CTGAC	6678/159720	6696/159738	6149/157382	6167/157400
DR7R	CGGGCCAGAACGAT AACAG	7051/160093	7033/160075	6514/157747	6496/157729
U18F	ATAACAGCATCGTA AATGCACCC	29505	29527	28567	28589
U18R	CATATCTGATCAACC TTGCGATG	30137	30115	29199	29177

Supplementary Fig S1. Phylogenetic analysis of HHV-6 DR regions.

A) Maximum-likelihood phylogenetic tree of DR region (without repeats) from 21 inherited-ciHHV-6B and two HHV-6B reference genomes (HST and Z29) as shown in Figure 3. Scale bar 0.002 nucleotide substitutions per site. B) The phylogenetic network generated from the same dataset, but without HST, Z29, and ciHHV-6B genomes from HGDP00813 and HGDP00092. C) Maximum-likelihood phylogenetic tree of DR regions from 6 inherited-ciHHV-6A and four HHV-6A reference genomes (U1102, AJ, GS1 and GS2). Scale bar 0.005 nucleotide substitutions per site. D) A network of the HHV-6A DR regions.

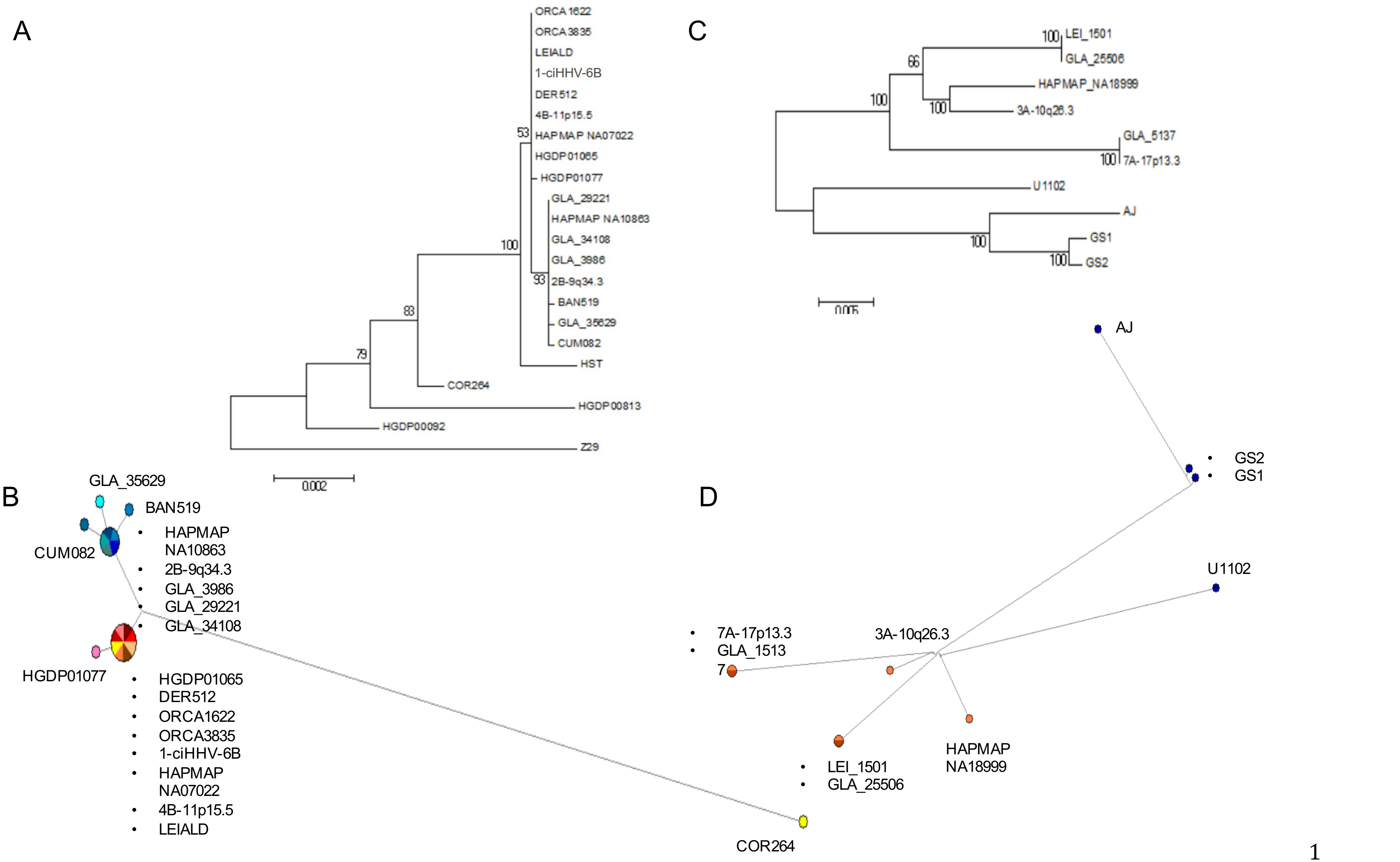

Supplementary Fig S2. Maximum-likelihood phylogenetic trees of individual genes from 21 inherited-ciHHV-6B and two HHV-6B reference genomes (HST and Z29). Scale bars are shown for each gene tree.

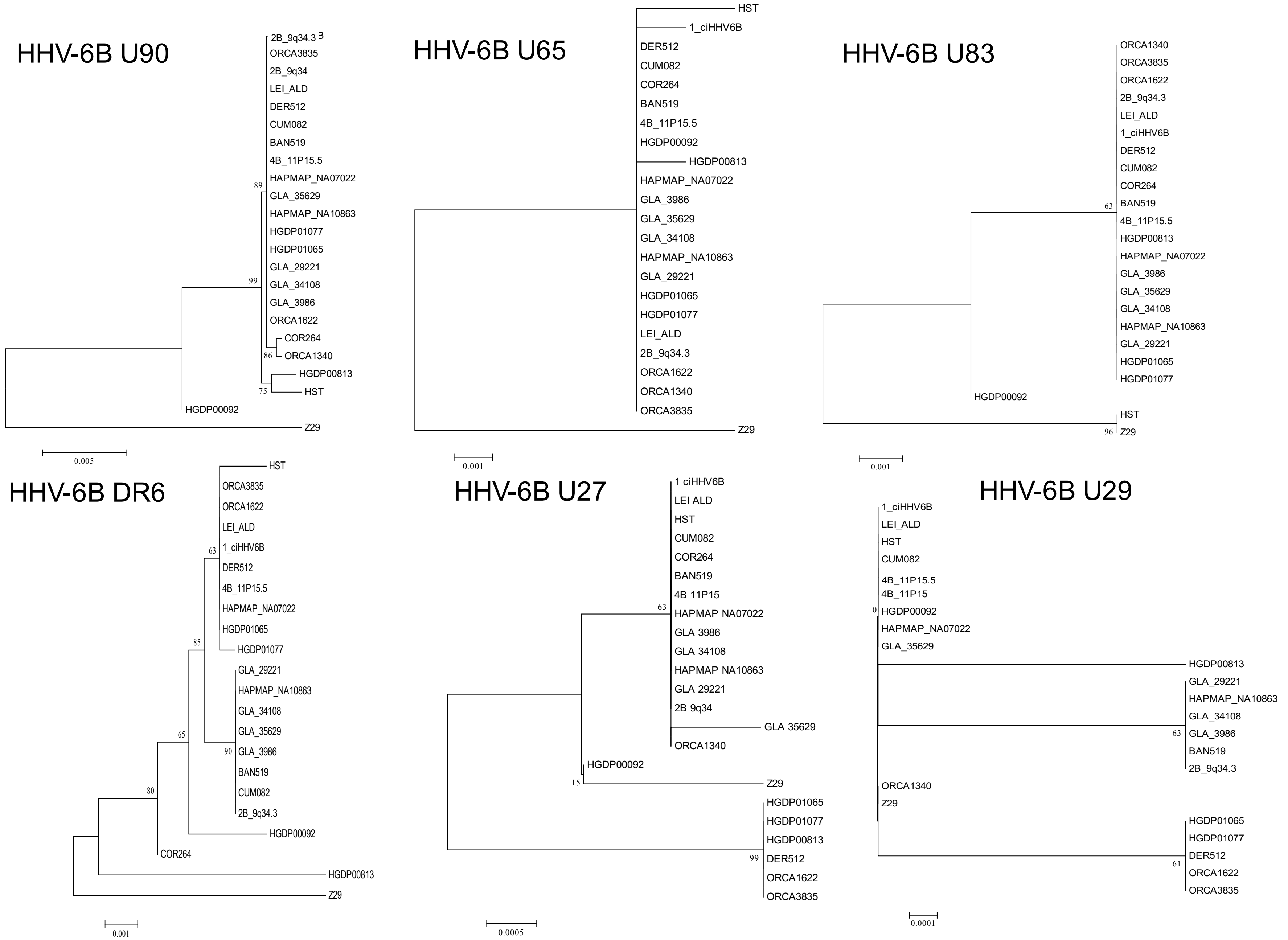

Supplementary Fig. S3. Maximum-likelihood phylogenetic trees of individual genes from seven inherited-ciHHV-6A and three HHV-6A reference genomes (U1102, AJ and GS). Scale bars are shown for each gene tree.

HHV-6A U83

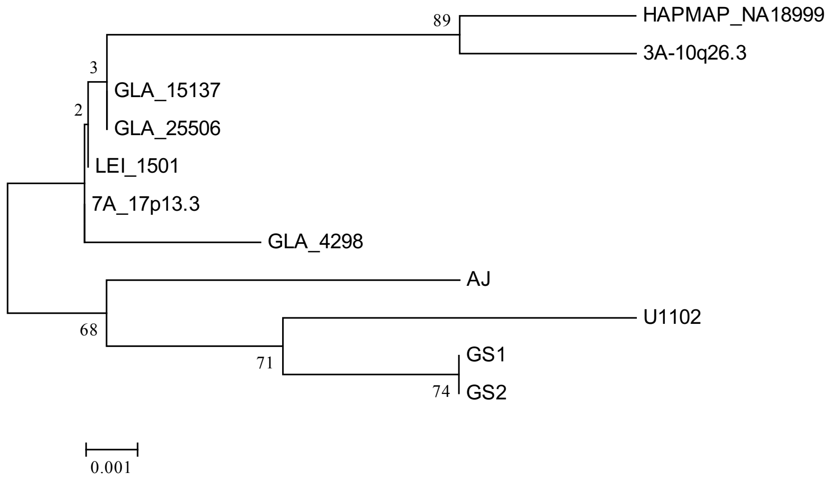

HHV-6A U90

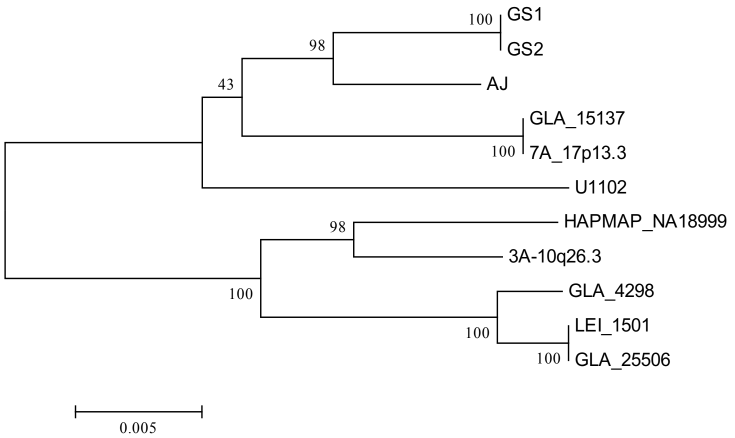

HHV-6A DR6

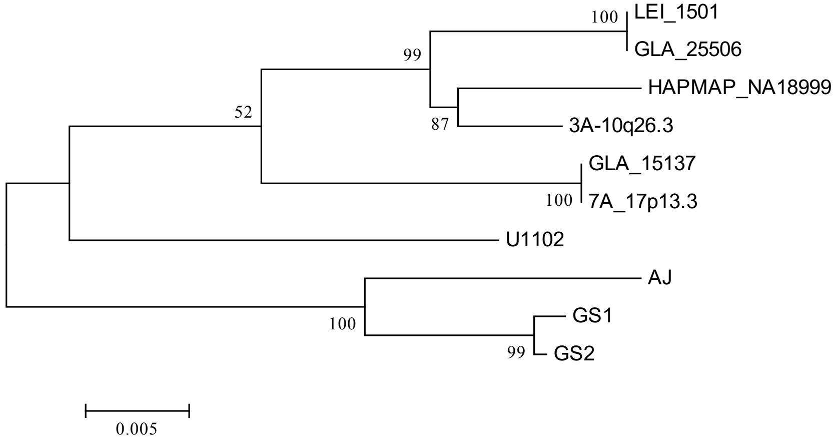

Supplement: Supplemental material [file JVI.01137-17_zjv999183050s1.pdf]
